# Improved Calibration of the Human Mitochondrial Clock Using Ancient Genomes

**DOI:** 10.1093/molbev/msu222

**Published:** 2014-08-05

**Authors:** Adrien Rieux, Anders Eriksson, Mingkun Li, Benjamin Sobkowiak, Lucy A. Weinert, Vera Warmuth, Andres Ruiz-Linares, Andrea Manica, François Balloux

**Affiliations:** ^1^UCL Genetics Institute, Department of Genetics, Evolution and Environment, University College London, London, United Kingdom; ^2^Department of Zoology, University of Cambridge, Cambridge, United Kingdom; ^3^Department of Evolutionary Genetics, Max Planck Institute for Evolutionary Anthropology, Leipzig, Germany; ^4^Department of Veterinary Medicine, University of Cambridge, Cambridge, United Kingdom

**Keywords:** Bayesian phylogenetic inference, mitochondrial substitution rates, divergence times, human, calibration strategy, ancient genomes, molecular clock

## Abstract

Reliable estimates of the rate at which DNA accumulates mutations (the substitution rate) are crucial for our understanding of the evolution and past demography of virtually any species. In humans, there are considerable uncertainties around these rates, with substantial variation among recent published estimates. Substitution rates have traditionally been estimated by associating dated events to the root (e.g., the divergence between humans and chimpanzees) or to internal nodes in a phylogenetic tree (e.g., first entry into the Americas). The recent availability of ancient mitochondrial DNA sequences allows for a more direct calibration by assigning the age of the sequenced samples to the tips within the human phylogenetic tree. But studies also vary greatly in the methodology employed and in the sequence panels analyzed, making it difficult to tease apart the causes for the differences between previous estimates. To clarify this issue, we compiled a comprehensive data set of 350 ancient and modern human complete mitochondrial DNA genomes, among which 146 were generated for the purpose of this study and estimated substitution rates using calibrations based both on dated nodes and tips. Our results demonstrate that, for the same data set, estimates based on individual dated tips are far more consistent with each other than those based on nodes and should thus be considered as more reliable.

## Introduction

Accurate estimates of mutation rates are crucial for a thorough investigation of the evolutionary history of virtually any species (Ho and Larson 2006; Ho et al. 2008), including ours (Scally and Durbin 2012). Because differences between the DNA of any two individuals correspond to mutations accumulated since their common ancestor, knowing the rate at which such changes arise allows estimating the time since divergence between any two stretches of DNA. This approach, referred to as “the molecular clock,” has been frequently applied to date key chapters in human evolutionary history, such as the dawn of humankind millions of years ago (Sarich and Wilson 1967; Hasegawa et al. 1985; Goodman 1999; Carroll 2003) or the expansion of anatomically modern humans (AMHs) from an African cradle some 100 k years ago (Stringer and Andrews 1988; Ingman et al. 2000; Stringer 2002; Cavalli-Sforza and Feldman 2003; Relethford 2008; Tattersall 2009). Mitochondrial DNA (mtDNA) has often been the marker of choice for this kind of investigations thanks to attractive characteristics such as high copy number, apparent lack of recombination, and high substitution rate (Ingman et al. 2000).

Accurately estimating substitution rates is not a trivial affair. For humans, different methodologies have produced disparate and sometimes irreconcilable estimates, thus raising doubts about the timescale of major evolutionary events (Ho and Endicott 2008; Endicott et al. 2009; Scally and Durbin 2012). Despite recent statistical advances allowing for fine modeling of rate heterogeneity among sites and lineages (Welch and Bromham 2005), further research is still needed to improve our confidence in molecular estimates of mutation rates (Hipsley and Muller 2014). In particular, the most reliable calibration points for converting molecular genetic divergences among branches into absolute times remain to be identified.

Phylogenetic rate estimates have historically been based on a single, external human–chimpanzee divergence calibration point at the root of the tree (Ovchinnikov et al. 2000; Tang et al. 2002; Mishmar et al. 2003; Soares et al. 2009), but recent evidence (Ho et al. 2005; Ho, Shapiro, et al. 2007; Ho and Endicott 2008) has strengthened previous suspicions (Stoneking et al. 1992; Bandelt et al. 2006) that calibration points within the human tree would be preferable (Bandelt et al. 2006; Stoneking et al. 1992). Consequently, several estimates of rates have been generated following the adoption of a series of nodes (branching points in the tree) calibrated with archaeological and/or biogeographic information (Atkinson et al. 2008; Endicott and Ho 2008; Ho and Endicott 2008; Henn et al. 2009; Pereira et al. 2010).

Even more recently, the increasing availability of sequence data from ancient DNA (aDNA) (Green et al. 2008; Krause, Briggs, et al. 2010; Fu, Meyer, et al. 2013; Fu, Mittnik, et al. 2013) has allowed phylogenetic trees to be calibrated from their tips (Rambaut 2000; Drummond et al. 2002). As a first attempt, Krause, Fu, et al. (2010) combined the use of the human–chimpanzee divergence calibration with tip calibrations from whole-genome sequencing of two extinct hominids (one Neanderthal and one Denisovan 38,310 and 40,000 years old, respectively). Interestingly, their results indicate that the estimated divergence times are largely dominated by the prior assumption on the root and are not sensitive to the two external tips. More recently, Brotherton et al. (2013) and Fu, Mittnik, et al. (2013) both made use of two distinct panels of ancient AMHs (aAMHs) whole-mtDNA sequences to perform the first estimations of mtDNA substitution rates from tip calibrations alone.

Previously published phylogenetic rates are not readily comparable because they were generated from different sets of sequences, used different genes or mitochondrial-genome sections, or assumed different combinations of calibration methods (root-, node-, and tip based). Additional statistical methodological differences between studies include 1) consideration of rate heterogeneity among sites and lineages, 2) correction for multiple substitutions at the same site (i.e., saturation), 3) incorporation of uncertainty around the age of calibration points, and 4) different demographic models. All the factors listed above are known to potentially influence substitution rate and timescale estimates (Welch and Bromham 2005; Drummond et al. 2006; Ho and Larson 2006; Henn et al. 2009; Nielsen and Beaumont 2009; Sauquet et al. 2012; Molak et al. 2013).

In this study, we compared rates and timescale estimates obtained using various calibration approaches (root, internal nodes, and tips) but using the same data set and statistical framework. To this aim, we assembled a large, worldwide data set of 30 high-quality ancient and 320 contemporary whole human mtDNA genomes. Of the contemporary sequences, 146 were generated for the purpose of this study.

## Results

### Sequence Composition

We found no evidence for differences in base composition between contemporary AMH (cAMH) and aAMH (unpaired *t*-test *P* value = 0.890). Ancient archaic humans (aAH) displayed a slightly, albeit significantly lower frequency in GC content than the aAMH and cAMH (unpaired *t*-test *P* value = 0.044) (supplementary appendix S5, Supplementary Material online).

### Damage Patterns in aDNA Sequences

A total of 1,973 single-nucleotide polymorphisms (SNPs) were called from the alignment including all human sequences but excluding the chimpanzee. No triallelic SNPs were found in the ancient sequences (aAMH + aAH). Among the biallelic SNPs, we specifically investigated patterns of singleton SNPs to detect any signal of damage in ancient sequences. As detailed in supplementary appendix S6, Supplementary Material online, we found that four ancient sequences (Ire8, Ajv52, Ajv70, and Gok4, see supplementary table S3, Supplementary Material online, for details on those sequences) exhibited a substantial excess of A/T (and deficit of G/C) singleton SNPs, suggesting an effect of deamination. When excluding those sequences, numbers of A/T singleton SNPs among the remaining sequences were not different from expectations computed assuming no aDNA damage. On this basis, we excluded those four ancient sequences from all subsequent analyses.

### Phylogenetic Analyses

The optimal partitioning scheme and the best-fit nucleotide substitution model for each partition of the mtDNA molecule were estimated using the software PartitionFinder (Lanfear et al. 2012). The best partitioning was obtained for *K* = 4 schemes (PC1+2, PC3, HVS1+2, and r+tRNA, see supplementary appendix S7, Supplementary Material online, for details) and was used henceforth. All phylogenetic analyses were performed with BEAST 1.7.4 (Drummond and Rambaut 2007). To determine the most appropriate demographic model for the human clade, we first used a data set containing only AMHs. The exponential growth model performed better than the two other models tested (Bayes factor of 16.51 and 25.91 compared with the logistic growth and the constant population size model, respectively) and was henceforth used for all subsequent analyses. Then, we tested for the best molecular clock model. The uncorrelated lognormal relaxed clock (UCLN) performed better than the strict clock (SC) (Bayes factor of 67.5 and 69.2 using the path sampling [PS] and stepping-stone sampling [SS] method to estimate marginal likelihoods, respectively) and was used henceforth.

Finally, we tested whether considering a model of DNA postmortem damage (PMD) in BEAST (Rambaut et al. 2009) would significantly affect substitution rate estimates. Accounting for PMD (while assuming tip-based calibration) did not significantly increase the marginal likelihood of the model (Bayes factor of 3.8 and 4.5 using the PS and SS methods, respectively). The rate of estimated damage was extremely low: 1.03 × 10^−^^9^ errors/site/year (95% highest posterior distribution [HPD]: 1.91^−^^14^–2.60^−^^9^), which translates into an expectation for the oldest (hence most damaged) AMH sequence (sample Tianyuan; 39,464 years old) of only 0.67 deaminated sites. Finally, accounting for PMD had a limited influence on the rate estimation. We obtained a value of 2.23 × 10^−^^8 ^µ/site/year (95% HPD: 1.71–2.75 × 10^−^^8^) for the whole molecule, which is 1.04× (4.2%) higher than when not accounting for PMD.

### Calibrating the Human mtDNA Tree

We compared the effect of different tree calibration strategies on the Bayesian estimation of substitution rates and divergence times. In BEAST, priors were placed on tips, internal nodes, or the root, as detailed in the Materials and Methods section. Date-randomization tests (Firth et al. 2010) indicated that the temporal signal present in the panel of ancient sequences is informative enough to perform a thorough estimation of substitution rates: Rates estimated on the ten randomized data sets do not overlap with those from the original data set (see supplementary appendix 8).

#### The Effect of Tip-Dating Errors

In BEAST, we tested the influence of incorporating uncertainty in ancient sample ages on posterior samples. As expected, incorporating the uncertainty in sample ages reduced the precision of estimates (i.e., the analysis taking uncertainties into account leads to higher standard error in the mean value of all parameters). Although this effect was small (the 95% HPD intervals of the posterior rate estimates changed by ≈4%, 5%, and 3% for the time to most recent common ancestor [TMRCA] of AMH, the human–chimpanzee divergence time, and the substitution rate estimate for the whole molecule, respectively), all the median values differed significantly between the parameters estimated with and without taking uncertainty in tip dates into account (Wilcoxon signed-rank test *P *value < 2.2E-16 in all cases). Ages were systematically older (and rates smaller) when accounting for sample age uncertainty. On this basis, all subsequent analyses took date uncertainty into account.

#### Tree Topology

**Fig. 1.**
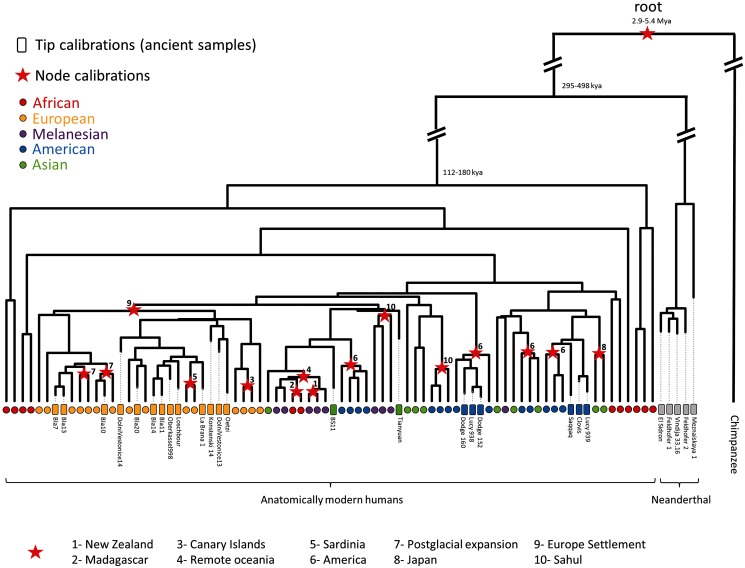
Phylogenetic tree built on a subset of both contemporary and ancient samples. This phylogeny was constructed with the BEAST software using 59 cAMHs, 21 aAMHs, and 5 archaic Neanderthals. The topology presented is well resolved as all nodes exhibit a posterior probability greater than 0.9. The end depth of branches for ancient samples reflects their ages. Ancient and contemporary samples are indicated with rectangles and circles, respectively, and colors indicate their geographical origin. Date estimates for major divergence events estimated using tips calibration are shown at the nodes. The number next to the red stars indicates the colonization/migration event associated with the node.

We found no significant differences between the number of topological differences (calculated using the Penny and Hendy [1985] statistic) within and between trees obtained assuming different calibration scenarios (Mann–Whitney *U* test *P *value = 0.678). This result suggests that, in our analyses, the calibration scenario does not significantly influence the topology. The best-supported tree (extracted using TreeAnnotator while performing tip calibration) is given in supplementary appendix S9, Supplementary Material online (but see fig. 1 for a tree based on a reduced subset of 85 sequences). We found a positive and significant relationship between the ages of the sequences and root-to-tip distances (supplementary appendix S10, Supplementary Material online). This pattern illustrates the gradual increase of DNA sequence variation over the sampling time interval and confirms that ancient samples do not exhibit any suspicious number of sequencing and/or dating errors. Finally, the TMRCAs of the major haplogroups present in our data set (generated assuming tip-based calibration) are reported in supplementary appendix S11, Supplementary Material online.

#### The Effect of Calibration Scenario on Bayesian Parameter Estimates

**Fig. 2.**
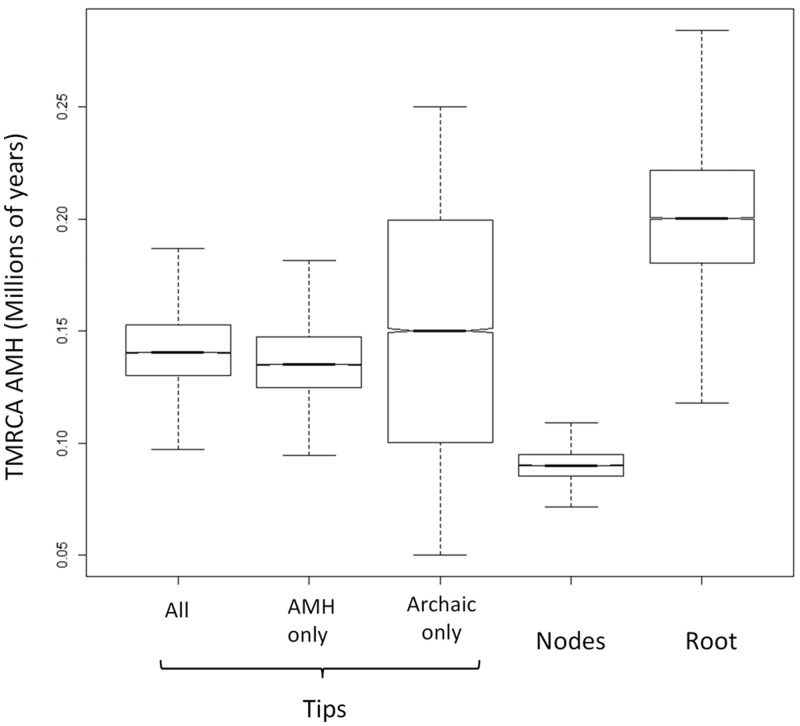
Estimates of the TMRCA of all AMHs. Box plot (minimum, quartiles, median, and maximum) for Bayesian estimates of the TMRCA of all modern humans (in Ma) obtained under various calibration scenarios.

We found significant (all unpaired *t*-tests, *P *value < 0.05) and substantial variation in the estimated divergence times and whole-molecule substitution rates among the different calibration scenarios (see fig. 2 and supplementary appendix S12, Supplementary Material online). Root calibration systematically yielded the smallest substitution rates (and oldest divergence times) estimates. Conversely, the highest substitution rates (and smallest divergence times) were produced by internal node calibration. Tip calibration displayed intermediate values between those two extremes (table 1 and supplementary appendix S12, Supplementary Material online). Interestingly, extra-specific tip calibration using archaic human samples generated overdispersed 95% HPD intervals for all parameters. Intraspecific tip calibration (aAMH only) yielded substitution rate estimates very similar to those obtained using all ancient samples (aAMH + aAH).

**Table 1.** Substitution Rate and Divergence Time Estimates Obtained with Tip Calibration Using All Ancient Samples.

Statistic	Divergence Times (Ma)			Substitution Rates (µ/Site/Year) (Units of 10^−8^)					SC Deviation (ucld.stdev)				
	Human– Chimpanzee	TMRCA AMH	AMH– Neanderthal	PC1 + PC2	PC3	HVS1 + HVS2	R + t RNA	Whole mtDNA	PC1 + PC2	PC3	HVS1 + HVS2	R + t RNA	Whole mtDNA
Best estimate	4.140	0.143	0.389	0.756	3.323	31.434	1.007	2.143	0.145	0.336	0.327	0.102	0.209
95% HPD	2.991	0.112	0.295	0.571	2.568	22.560	0.757	1.580	0.000	0.247	0.186	0.000	0.116
	5.448	0.180	0.498	0.935	4.074	40.306	1.266	2.706	0.289	0.428	0.465	0.253	0.297

#### Variation among Individual Internal Node and Tip Calibration

**Fig. 3.**
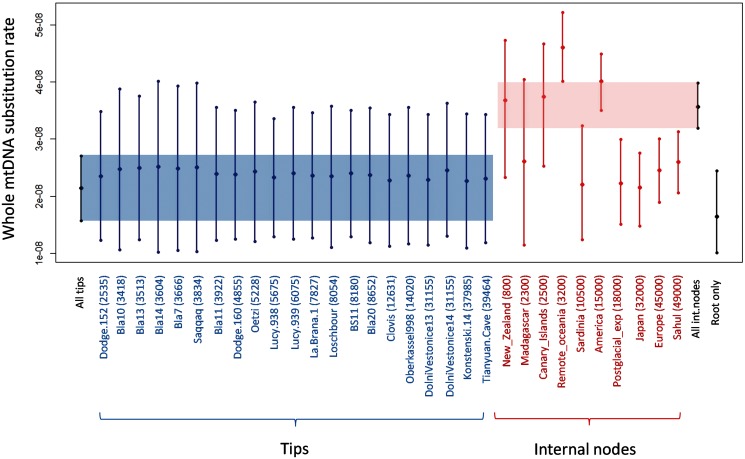
Variation in substitution rate among individual calibrations. Substitution rate estimates obtained using individual tip and node calibrations are reported for the whole mtDNA (in µ/site/year). Results are reported as 95% HPD intervals. Individual tips and nodes are sorted by their respective ages (given in years in brackets, see supplementary appendices S3 and S4, Supplementary Material online, for details). Global estimates obtained using all tips or all internal nodes simultaneously for calibration, as well as root-based estimate are also given for comparison.

To test whether the inferred substitution rates depend on any one dated tip or internal node, we performed BEAST analyses using each ancient mtDNA sequence and each colonization/migration event independently as a separate calibration point. We found substantial variation in substitution rate estimates obtained using different tips/nodes (fig. 3 and supplementary appendix S13, Supplementary Material online). The 95% HPD interval obtained for each independent ancient sequence systematically overlapped with all other HPDs, as well as with the mean estimate generated considering all tips simultaneously, suggesting that no single ancient sample is driving the overall substitution rate estimate. The situation is strikingly different for individual internal node calibration. Indeed, the mean estimate generated considering all internal nodes simultaneously overlaps with only four (out of ten) of the 95% HPD interval obtained with each individual node. Moreover, the variance observed among individual substitution rate estimates was 11 times larger for internal nodes than tips. Altogether, these results suggest that tip calibration yields far less dispersed and more consistent estimates than internal node calibration.

We also detected a subtle effect of time dependency on rate estimates, with older calibration points leading to slower substitution rates (Ho, Shapiro, et al. 2007; Ho, Lanfear, Bromham, et al. 2011). We observed a negative linear correlation between the age of the calibration point (tips and internal nodes) and the substitution rate inferred at the whole-mtDNA molecule (*r* = −0.53, see supplementary appendix S13, Supplementary Material online).

### Deviation from a “Strict Clock”

We tested for deviations from a strict molecular clock. First, we observed different values of ucld.stdev (the standard deviation of the uncorrelated log-normal relaxed clock) for the different partitioning schemes among the different calibration scenarios (supplementary appendix S12, Supplementary Material online). The highest estimate was 0.358 (for root calibration on the PC3 subset), thus suggesting very little variation (ucld.stdev value < 1) in rates among branches. Second, to empirically investigate whether the small deviation from an SC could be due to rate heterogeneity in the ancient branches (those leading to the chimpanzee and archaic humans), we performed additional BEAST analyses considering either only Hominin sequences (without the chimpanzee) or only modern human sequences (without the chimpanzee + archaic humans) while applying tip calibration. In both cases, the relaxed clock still performed better than the SC (Bayes factor of 61.3 and 47.8 obtained using the PS method to estimate the marginal likelihood for analyses excluding the chimpanzee and the chimpanzee + archaic humans, respectively), thus indicating no major influence of the archaic humans and the chimpanzee sequences.

**Fig. 4.**
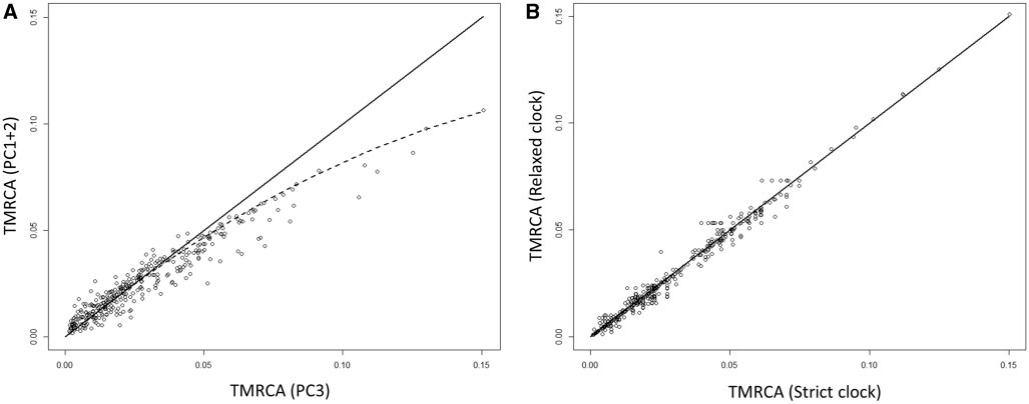
TMRCAs of AMHs obtained under different phylogenetic parameters. TMRCAs (in Ma) between all pairs of samples (belonging to the AMH clade only) obtained under different phylogenetic reconstruction parameters. The topology of the tree obtained using the full data (all sites in the mtDNA, see supplementary appendix S9, Supplementary Material online) has been fixed in BEAST. Panel (*A*) represents TMRCAs estimated the PC3 versus PC1+2 subsets of nucleotides. The solid line shows *y* = *x* line, and the dashed line is a polynomial regression of degree 2. Panel (*B*) represents the age of all nodes when independently estimated using either a strict or relaxed clock; the linear regression coefficient between the two sets of nodes is *R*^2 ^= 0.98.

To explain why the relaxed clock was a better fit than the SC, we used BEAST to investigate the effect of natural selection, more specifically the effect of purifying selection acting on the coding regions of the molecule. When plotting the TMRCAs between all pairs of sequences obtained for PC1+2 and PC3 sites independently, we observe a clear deviation from linearity, with older TMRCAs from about 30 ka showing a deficit of nonsynonymous sites mutations, relative to the primarily neutral mutations at the third position (fig. 4*A*). An acceleration of substitution rates in recent times has been previously documented for mtDNA and has been interpreted as reflecting the time needed for purifying selection to purge slightly deleterious mutations from the genome (Soares et al. 2009). However, although the relaxed clock fits the data significantly better, the strict and relaxed rates over the whole molecule are extremely close to each other and essentially interchangeable in the context of phylogenetic dating of human evolutionary events. This is illustrated by the extremely tight 1:1 relationship observed between the TMRCAs estimated between all pairs of sequences with the strict- versus relaxed clock (*R*^2 ^= 0.98; fig. 4*B*).

## Discussion

In this study, we investigated the influence of the calibration strategy on estimates of human mitochondrial substitution rates and divergence times using whole mitochondrial genomes of 320 modern and 26 ancient samples. Comparisons of tip- versus node-based phylogenetic estimates have been performed in a number of other species before (Ho, Kolokotronis, et al. 2007; Gilbert et al. 2008; Ho et al. 2008). However, they all relied on comparisons of internal (radiocarbon ages of ancient sequences) to external (split between the species under study and an outgroup) calibration points. Because of its detailed archaeological history, humans offer a unique opportunity to compare calibration points based both on internal tips and nodes over a comparable time-scale.

Our results show that the calibration strategy has no significant influence on the reconstructed topology but has a notable effect on substitution rate and divergence time estimates. Despite using the same analytical steps on a single data set, we obtained slower substitution rates (by a factor of 0.65-fold) for tip-based estimates (compared with node-based ones, see supplementary appendix S12, Supplementary Material online). This result is at odds with observations made in numerous other species where the use of aDNA sequences yielded faster rate estimates (Ho, Kolokotronis, et al. 2007; Ho, Shapiro, et al. 2007; Millar et al. 2008; Ho, Lanfear, Bromham, et al. 2011). The faster rate for calibration based on ancient sequences has been explained by the “time-dependency of molecular rates” hypothesis, which postulates an acceleration over recent times in coding sequences due to the time needed for selection to purge slightly deleterious mutations (Ho, Lanfear, Bromham, et al. 2011). However, in our study the range of ages for calibrated nodes and tips largely overlaps. This impede any theoretical prediction on which rates should be faster. Internal nodes may nevertheless lead to a systematic overestimation of rates if genetic divergence precedes population divergence, as discussed by Peterson and Masel (2009).

Our tip-based estimates of substitution rates are highly consistent with the values recently published by Brotherton et al. (2013) and Fu, Mittnik, et al. (2013) who used two very different panels of aDNA sequences to calibrate the human mtDNA tree. Consistently with the differences between tip- and node-based calibrations, we report here, our tip-based rate estimates are slower (by a factor of 0.63-fold) than the ones obtained using internal node calibration by Endicott and Ho (2008) but are faster (by a factor of ∼1.5-fold) than previous fossil-calibrated rates (Green et al. 2008; Soares et al. 2009). Although we cannot know which of these estimates are closest to the value of the “real” long-term substitution rate of human mtDNA, we can evaluate which calibration approach leads to the most consistent estimates. The answer to this question is unambiguous; the variance over individually calibrated substitution rates is 11 times smaller for tips than internal nodes. Moreover, all of the 21 substitution rates estimated from aAMH sequences had overlapping 95% HPD (fig. 3). The situation is strikingly different for node-based calibrations, where substitution rate estimates strongly depended on the demographic episode used for dating, with only four out of ten individually calibrated rates having overlapping HPDs.

These results strongly suggest that tip calibration estimates are far more consistent than internal node-based ones. However, tip-based calibration also point to slower mean substitution rates than those based on internal nodes. Thus, one important question we need to answer is whether tip-based calibrations are affected by some systematic bias that might lead to slow (and homogeneous) substitution rates. Such potential systematic bias could arise for various reasons, including 1) sequencing errors and/or PMDs in ancient sequences (Ho et al. 2005; Ho, Heupink, et al. 2007; Ho, Kolokotronis, et al. 2007; Rambaut et al. 2009), 2) data sets with low information content (Debruyne and Poinar 2009; Ho, Lanfear, Phillips, et al. 2011), 3) demographic and/or genetic model misspecification (Emerson 2007; Navascues and Emerson 2009; Ho, Lanfear, Bromham, et al. 2011), and 4) other factors such as a clumped distribution of ancient sample ages (Ho, Lanfear, Bromham, et al. 2011; Ho, Lanfear, Phillips, et al. 2011). However, we feel confident that none of the sources of bias listed above is affecting our rate estimates.

A bias generated by sequencing errors and/or PMDs in the ancient samples seems unlikely for a series of reasons. First, the sequences were generated by several laboratories, using different sequencing technologies and post processed considering different contamination assays. The read depth of all the sequences included is high, thus minimizing the risk of incorrectly called SNPs. Second, our new method devised to detect an excess/deficit of deaminated singletons suggests that none of the sequences which were included was affected by detectable levels of postmortem DNA damage. This was confirmed by the application of the PMD model in BEAST, which did not influence significantly the estimation of the substitution rates.

Our ancient sequence age randomization test clearly indicated that our data set contains a strong enough signal to accurately calibrate the reconstructed human mtDNA phylogeny. This, combined with the fact that convergence was systematically achieved in BEAST, disqualifies both the hypothesis of “low information content” and “restricted ancient sample age distribution.” Finally, we did not detect any significant influence of varying demographic models and prior choices on our Bayesian inferences (results not shown), which argues against the remaining listed systematic biases. Moreover, confidence in our tip-based estimates is bolstered by the results from two recent independent analyses (Brotherton et al. 2013; Fu, Mittnik, et al. 2013). Both articles report very similar tip-based rate estimates to ours despite using different genetic/demographic models in BEAST, as well as sequences from different ages and geographic origins. Taken together, all the available evidence suggests that tip-based calibration of human mtDNA are fairly immune to systematic biases and constitutes a strong case for them to be the most biologically relevant.

The superior performance of tip calibration over node calibration is not entirely surprising when we consider the various sources of uncertainties associated with both calibration strategies. The uncertainty associated with tip-dating simply mirrors the uncertainty in the estimated age of the sequences—in this case, the error of the C-14 radiocarbon dating technology (Bowman 1990; Molak et al. 2013). This is a well-characterized source of error which can and should be integrated into phylogenetic inference (Drummond et al. 2006; Ho and Phillips 2009), even if most recent studies still rely on point values (Krause, Fu, et al. 2010; Fu, Mittnik, et al. 2013).

In contrast, the uncertainty around dated nodes is far more complex and multifactorial and is likely to lead to different degrees of reliability associated to each node. First, there is generally considerable uncertainty associated with the age of the colonization/migration event including error in the dating of the archaeological, anthropological, and historical evidence. The age of the oldest evidence for human presence is unlikely to coincide exactly with the demographic expansion. Very generally, we would predict to see a delay in the appearance of traces of human presence after the expansion of AMHs into any new area (Signor and Lipps 1982). However, we could also think of cases where the initial settlement which led to the archaeological evidence was followed by local population extinction and thus predates the age of the of the clade in the phylogenetic reconstruction; such a scenario has been suggested for the Qafzeh–Skhul early AMHs who are generally believed to have been part of an early, failed out-of-Africa exit (Oppenheimer 2012).

Second, even if a demographic event had been accurately dated, the age of the node in the phylogenetic tree might not coincide with it for a number of reasons (Edwards and Beerli 2000; Ho and Phillips 2009; Balloux 2010; Firth et al. 2010; Crandall et al. 2012). For instance, the phylogenetic node of interest may correspond to the most recent common ancestor (MRCA) of the sampled sequences rather than the split of the population of interest. But even if the population was accurately sampled, we might still envision cases where the age of the coalescence does not coincide with the demographic event. Such a mismatch could arise if the founding population was already polymorphic for the marker under study, so that the estimated coalescence event is older than the population split (i.e., incomplete lineage sorting). A similar situation could arise when multiple waves of colonists contributed to a demographic expansion. Conversely, the population might have experienced a reduction in size later on, so that the TMRCA could coincide with this subsequent population bottleneck. We could think of additional scenarios and the situation would become even more complex if we considered a possible effect of natural selection. To summarize, node calibration can be affected by many sources of error, and it is thus nearly impossible to model the age uncertainty around nodes satisfyingly.

There is an extensive debate in the literature on the existence and significance of time-dependent rates of molecular evolution (Ho, Shapiro, et al. 2007; Ho, Lanfear, Bromham, et al. 2011). This acceleration in substitution rates in recent generations has previously been described and discussed in several species (Ho, Shapiro, et al. 2007; Ho, Lanfear, Bromham, et al. 2011; Duchene et al. 2014), including humans (Henn et al. 2009). It is generally ascribed to the time needed for natural selection to weed out slightly deleterious mutations from the population (Endicott et al. 2009; Soares et al. 2009). We observed a subtle but significant negative linear correlation between the age of the ancient sequence used for calibration and the substitution rate estimated (supplementary appendix S13, Supplementary Material online), which is consistent with this prediction. Stronger evidence for purifying selection comes in the form of the stark difference in the substitution rates of first and second codons (PC1+2) versus third codon (PC3) (fig. 4*A*). Although PC3 mutations accumulate linearly with time, we see a clear acceleration in the rate starting at around 30 ka for the mostly nonsynonymous mutations at PC1+2.

There has also been a vigorous debate in the literature regarding the extent to which positive selection and local adaptation may have shaped the current distribution of human mitochondrial, with a particular focus on the possible role of climate (Mishmar et al. 2003; Kivisild et al. 2006; Balloux et al. 2009; Soares et al. 2012). The fact that a relaxed clock fits our data best would be in line with the idea that rate variation in human mtDNA is subject to positive selection. Alternatively, this variation in evolutionary rates could stem from purifying selection on mtDNA being more effective in some human populations than others due to differences is population size and past demography. However, the effect we detect is extremely subtle and only statistically significant thanks to the large data set. We also failed to find any ecological or demographic factor that could explain variation in inter-individual rate variation satisfactorily (results not shown). Strict and relaxed rates are extremely close, and the distribution of TMRCAs for all pairs of sequences estimated with either rate are essentially interchangeable (*R*^2 ^= 0.98; fig. 4*B*) and would lead to near identical results in the context of using either rate for dating past human demographic events.

Using both anatomically modern and archaic ancient mtDNA sequences to calibrate the tree, we obtain a rate of 0.75 × 10^−^^8^, 3.32 × 10^−^^8^, and 2.14 × 10^−^^8^ substitutions per site per year for the coding region PC1+2, PC3, and the whole molecule, respectively. The rates changed little when we ignored the external calibration tips (archaic humans). This result points to external calibration tips having limited influence on rate estimates, as previously discussed by Krause, Fu, et al. (2010).

Using tip calibration, we also estimated the coalescence dates of various nodes of interests in the tree. We obtained a value of 4.1 Ma (95% HPD: 2.9–5.4) for the divergence between Hominins and chimpanzee. This estimation may appear too young when compared with the dates that are generally derived from the fossil record. The Toumaï fossil (*Sahelanthropus tchadensis*), dated to 6–7 My (Brunet et al. 2002), is usually interpreted as being on the Hominin line and setting a minimum date for the divergence (Vignaud et al. 2002). The apparent conflict between our tip-based estimation and the fossil record could be explained if Toumaï were somewhat younger than previously reported (Brunet et al. 2005) or if we assumed more complex speciation scenarios where an initial split was followed by an extended period of gene flow before the final separation, as suggested by Patterson et al. (2006).

We estimated a split time between *Homo neanderthalensis* and *H. sapiens* mtDNAs of 389 ka (95% HPD: 295–498). This is consistent with the 407 ka (95% HPD: 315–506) estimates of Endicott et al. (2010) which used the radiocarbon dates of the same five Neanderthal genomes but younger than the 550 ka (95% HPD: 496–604) estimates of Soares et al. (2009) obtained assuming a “6.5 Ma human–chimpanzee divergence”-based calibration. Considering the widely held view that *H. neanderthalensis* evolved from *H. heidelbergensis* in Europe (Sawyer et al. 2007), one would expect the appearance of *H. heidelbergensis* in Europe to predate the time of the split between the two lineages. The Boxgrove tibia (from Sussex, England), attributed to *H. heidelbergensis*, dates to approximately 500 ka (Roberts et al. 1994), which would be in line with our estimate for the timing of the split and this interpretation of the fossil record.

Our estimate of 143 ka (95% HPD: 112–180) for the TMRCA of all modern human mtDNA is slightly younger but highly consistent with the 157 ka (95% HPD: 120–197) value obtained by Fu, Mittnik, et al. (2013). We estimate the coalescence of the L3 haplogroup (the lineage from which all non-African mtDNA haplogroups descend), often used to date the “out-of-Africa” event, to 72 ka (95% HPD: 54–93), a value also consistent with Fu, Mittnik, et al. (2013) estimation of 78 ka (95% HPD: 62–95). This estimation rather places a conservative upper bound of 93 ka for the time of the last major gene exchange between non-African and sub-Saharan African populations. As pointed out by Fu, Mittnik, et al. (2013), it is important to recognize that this divergence time may merely represent the most recent gene exchanges between the ancestors of non-Africans and the most closely related sub-Saharan Africans and thus may reflect only the most recent population split in a long, drawn-out process of population separation (Scally and Durbin 2012).

Finally, our results also allowed us to check whether the coalescence dates of some major haplogroups associated with human migrations (table 2) are consistent with the archaeological evidence (supplementary appendix S14). For six out of ten of the colonization/migration events considered (Postglacial expansion, Sahul, Sardinia, Japan, Madagascar, and Europe settlement), we observed 95% age HPD distributions of haplogroups overlapping with the archaeological dates. However, in the case of the Canary Islands, Remote Oceania, New Zealand, and the Americas, the estimated coalescence times were systematically older than the archaeological evidence. Potential explanations for such discrepancies include ancestral polymorphism in the founding population or complex demographic histories involving multiples waves of colonists.

**Table 2.** Coalescence Times for Major Haplogroups Involved in the Colonization/Migration Events Considered.

Colonization/Migration Event	Haplogroup	Time Estimate (ka)		
		Mean	95% HPD	
Canary	U6b1 and U6b1a	9.72	4.55	15.80
Remote Oceania	B4a1a1a and B4a1a1a1	15.83	10.61	21.52
Sahul	P	56.77	43.37	70.91
Sardinia	U5b3a1a	10.80	5.33	16.71
New Zealand	B4a1a1a3	4.94	2.02	8.28
Japan	M7a	30.82	20.52	42.33
Madagascar	B4a1a1a2	4.97	1.25	9.32
Europe	U5-U6 split	50.38	38.67	62.06
America	A2	30.08	21.87	39.66
	B2	23.27	16.09	31.21
	C1	31.32	22.17	41.43
	D1	30.79	21.85	40.41
	X2a	21.43	14.15	29.52
	Mean	27.38	21.20	34.36
Postglacial expansion	H1	18.14	11.30	25.78
	H3	17.44	10.90	25.11
	Mean	17.79	12.06	24.20

Note.—Time estimates were obtained using tip calibration (based on all ancient samples).

In conclusion, our results demonstrate that the recent availability of ancient high-quality mtDNA genomes offers a powerful tool to robustly date past evolutionary events of our own species. Using the age of ancient sequences leads to far more reproducible inferences and allows circumventing the large number of assumptions behind node and root calibration, which in turn should lead to an improvement of the estimation of human mitochondrial substitution rates. It should be possible to obtain increasingly narrow and precise substitution rate estimates by including additional ancient genomes in the analyses, as they will become available. In this context, ancient isolates from geographic regions which are not represented yet, such as Africa and Australia, would be particularly helpful, as these would allow fine calibration of further clades in the human mitochondrial genome. From a more general point of view, the growing availability of ancient sequences due to sequencing technology improvements should allow reliable tip-calibrated phylogenetic rate and divergence time estimates to be obtained in many species for which internal nodes split times information are presently not available.

## Materials and Methods

### Data Set

Our data set composed of 320 cAMH and 30 ancient human complete mitochondrial genomes, consisting of both new and publicly available sequences.

#### New Sequences

A total of 146 new samples were obtained from two different sources. First, 102 samples were selected by randomly choosing two individuals from each of the 51 populations of the HGDP-CEPH human genome diversity cell line panel (Cann et al. 2002). Second, we randomly selected two individuals from each of the 21 Native American and one Siberian populations that had previously been genotyped at autosomal microsatellites (Wang et al. 2007), yielding a total of 44 samples. Whole-mtDNA genomes were sequenced for all 146 samples at an average coverage of 643× (range: 43×–3,550×). Details on the molecular and data processing are given in supplementary appendix S1, Supplementary Material online. The HGDP-CEPH mtDNA sequences are part of a wider sequencing effort and were deposited in GenBank under accession numbers KF450814–KF451871. The 44 Native American samples are specific to this publication and were assigned GenBank accession numbers KJ923805–KJ923848.

#### Previously Published Sequences

A total of 174 public cAMH sequences were selected from GenBank to complete the geographic coverage and the haplogroup spectrum of the 146 cAMH sequences generated in this project (supplementary appendix S2, Supplementary Material online). Previously published and radiocarbon (C14) dated aDNA sequences were also obtained from GenBank, including 25 aAMHs and 5 aAH (supplementary appendix S3, Supplementary Material online). A chimpanzee sequence (accession number HM068573) was used as outgroup. All sequences were first aligned to the revised Cambridge Reference Sequence (accession number NC_012920) using the MUSCLE program (Edgar 2004). This resulted in a 16,650-bp aligned sequences matrix in which each nucleotide has been annotated by matching to the Cambridge reference annotation file using an in-house R script (R Core Team 2013).

### Assessing Sequence Quality of the Ancient Samples

It is well documented that substantial chemical modifications of nucleotide bases can be introduced postmortem as a result of DNA damage (Paabo 1989; Sawyer et al. 2012). This feature has been used to distinguish between ancient damaged sequences and putative modern contaminants (Green et al. 2008; Krause, Fu, et al. 2010). Here, we took advantage of the random nature of DNA damage to test for sequence quality in the ancient samples. Deamination of cytosine residues in C/G base pairs, leading to miscoding of C/G base pairs to T/A base pairs, represents the primary type of damage to aDNA. Thus, damaged sequences should carry an excess of A/T over C/G base pairs. Moreover, given that deamination errors are expected to happen at random, the probability of observing the same error in multiple sequences is low. Thus, we have a testable prediction that damaged sequences will be characterized by an excess of low frequency A/T relative to G/C alleles (e.g., singletons).

### Partitioning the mtDNA Molecule

The optimal partitioning scheme and the best-fit nucleotide substitution model for each partition of the mtDNA molecule was estimated using the software PartitionFinder (Lanfear et al. 2012). We defined the following seven groups of nucleotides: 1) HVS1, 2) HVS2, 3, 4, 5) protein coding positions at 1st, 2nd, and 3rd codon, 6) tRNAs, 7) rRNAs and used PartitionFinder to analyze every scheme that includes those seven groups in any possible combination. We also used the same algorithm to independently select the best model of nucleotide evolution to apply to the whole molecule.

### Estimation of Substitution Rates and Divergence Times

Bayesian phylogenetic analyses were performed with BEAST 1.7.4 (Drummond and Rambaut 2007). In all analyses on the partitioned data, substitution and clock models were unlinked, whereas tree topology was assumed to be the same between schemes. We also performed separate phylogenetic inferences on the whole-mtDNA molecule.

In BEAST, we first used a data set containing AMHs only to test for the best demographic coalescent model to consider as tree prior. We compared constant size, logistic growth, and exponential growth models. Then, applying the best demographic model on the total data set, we compared SC and UCLN. Finally, we tested whether considering a model of DNA PMD in BEAST (Rambaut et al. 2009) would significantly affect substitution rate and divergence time estimates. We specified a model where the probability that any transition sites of the alignment remained undamaged is assumed to decay exponentially with sample age.

Model choice was based on the Bayes factors calculated from the marginal likelihoods, the latter being computed using both the PS and SS methods as recently recommended by Baele et al. (2012, 2013).

Substitution models were based on the best fit from Partition Finder, and rate variation among sites (both including and excluding invariant sites) was modeled with a discrete gamma distribution with four rate categories. Note that the XML-input file was manually modified to apply the exponential growth model to the AMH clade only. An independent constant population size model was applied to the Neanderthal clade as recommended by Briggs et al. (2009).

In BEAST, posterior distributions of parameters, including divergence times and substitution rates are estimated by Markov chain Monte Carlo (MCMC) sampling. For each analysis, we ran four independent chains in which samples were drawn every 5,000 MCMC steps from a total of 50,000,000 steps, after a discarded burn-in of 5,000,000 steps. Convergence to the stationary distribution and sufficient sampling and mixing were checked by inspection of posterior samples (effective sample size > 200). Parameter estimation was based on the samples combined from the different chains. The best supported tree was estimated from the combined samples using the maximum clade credibility method implemented in TreeAnnotator.

### Calibrating the Human mtDNA Tree

We compared the effect of different tree calibration strategies on the estimation of substitution rates and divergence times. Priors were placed on tips, internal nodes, and root age.

#### Tip Dating

Tip age was specified using reliable dates available for all high-quality ancient complete human mtDNA sequences available at the time. Ages ranged from 2,535 (±235) years old (Dodge 152 sample) to 39,464 (±639) years old for aAMHs (Tianyuan Cave specimen) and to 64,500 (±5,200) years old for the archaic Neanderthals (Meznaiskaya1 specimen). Dates were calibrated in years before present with the R package Bchron using the INTCAL09 data set. Uncertainty around the dating was modeled in BEAST using a normal prior with standard deviation equal to the standard error of the radiocarbon date (supplementary appendix S3, Supplementary Material online).

We first used date-randomization tests to determine whether the temporal and genetic information contained in the ancient sequences were sufficient for a thorough estimation of substitution rates. From the total data set (composed of 350 sequences), ages of the sequences were randomly shuffled ten times, and date-randomized data sets were reanalyzed with BEAST. If the mean estimate of the evolutionary rate is not included in any of the 95% highest posterior density (HPD) intervals of the rate estimates from the date-randomized replicates, then the ancient sequences can be considered to have sufficient temporal structure and spread (Firth et al. 2010). Point-calibrated tips analyses (i.e., using the midpoint of the age range only) were also run to investigate the effect of incorporating uncertainty in sample ages on posterior samples. To prevent the chain from becoming stuck on unrealistic inflated values, flat priors (uniform distributions) were applied for all other internal nodes and root ages (0.0645–50 My) as well as for substitution rates (10^−^^10^–10^−^^5^ subst/site/year). Tip dating analyses were performed while considering aAMHs (intraspecific calibration) and archaic humans (extra-specific calibration) either separately or simultaneously. Finally, to test whether the inferred substitution rates are dependent on a single-dated aAMH sequence, we performed BEAST analyses using each ancient mtDNA sequence independently as separate tip calibrations.

#### Internal Node Dating

Internal node calibration was conducted by specifying priors on the ages of some specific nodes in the tree. To avoid as much as possible any subjective bias, we enforced the following three criteria: 1) the anthropological evidence must correspond to a discrete expansion or migration, 2) at least one candidate haplotype has to be associated with the demographic event, and 3) the age of the event needs to be based on solid archaeological evidence. These fairly stringent conditions let us to ignore several demographic events such as the “out of Africa” exit, for which we were unable to find archaeological evidence, which would allow us to assign a date to the migration. We could identify ten different events which fitted all our criteria: The colonization of Sahul, Europe, Japan, America, Sardinia, Remote Oceania, Canary Islands, Madagascar, New Zealand, and the post glacial re-expansion of populations from the Franco-Iberian refuge areas (see supplementary appendix S4, Supplementary Material online, for details). We used normal distributions as priors on nodal ages with the mean being equal to the archaeological date of first settlement (or of re-expansion) and a standard deviation of 20% around the mean. Analyses were run considering all ten internal nodes either simultaneously or independently.

#### Root Dating

The root date was specified using a prior on the age of the human–chimpanzee split, which was assumed to follow a log normal distribution with a minimum of 5 My, a mean of 6 My, and with 95% of the distribution lying between 5 and 7.5 My. These values were chosen for consistency with previous studies and information from the fossil record (Benton and Donoghue 2007).

Equal amounts of computational time (number of MCMC iterations and independent chains as previously given) were employed for the different calibration scenarios. We also selected an equal number of posterior samples to estimate parameters and the marginal likelihood.

### The Effect of the Calibration Scenario on Phylogenetic Estimates and Topology

We first assessed the influence of taking into account uncertainty in tip dates by testing whether point and nonpoint calibrations give significantly different results for substitution rate and root age estimates (95% HPD intervals, Wilcoxon signed-rank non directional tests). Secondly, we investigated whether the calibration scenario had an influence on the tree topology. To do so, it is necessary to control for the MCMC error in the best supported topology that can be inferred among independent BEAST runs performed assuming the same calibration approach. Topological distances between pairs of trees (calculated on a sample of ten independent trees inferred per calibration scenario) were generated using the Penny and Hendy statistic (Penny and Hendy 1985), which is defined as twice the number of internal branches defining different bipartitions of the tips. To be comparable between all scenarios, trees were pruned to contain AMH tips only. Finally, the effect of the calibration scenario on the divergence time and substitution rate estimates was assessed by comparing 95% HPD interval sizes using pairwise independent-samples *t*-test.

### Strict versus Relaxed Clock

We tested for deviations from a strict molecular clock by considering the standard deviation of the uncorrelated log-normal relaxed clock (the parameter ucld.stdev) obtained for the different schemes. If this parameter is small (close to 0), there is little variation in rates among branches, and the data are clock like. If this parameter takes values greater than 1 then the standard deviation in branch rates is greater than the mean rate, and the data exhibit substantial rate heterogeneity among lineages. We also empirically investigated the effect of the chimpanzee and archaic human sequences on rate heterogeneity among lineages. To do so, we ran two analyses using alignments excluding those sequences (one excluding the chimpanzee only and one excluding both the chimpanzee and Neanderthal sequences) and performed model comparison through the evaluation of Bayes factors, as described previously.

## Supplementary Material

Supplementary appendices S1–S14, tables S3–S7 and S11–S13, and figures S2, S5, S8, S10, and S12–S14 are available at *Molecular Biology and Evolution* online (http://www.mbe.oxfordjournals.org/).

Supplementary Data
