# Supplementary material for: Improved Calibration of the Human Mitochondrial Clock Using Ancient Genomes
Source: Mol Biol Evol. 2014 Aug 5;31(10):2780–92. doi: 10.1093/molbev/msu222 (PMC4166928; doi:10.1093/molbev/msu222)
Supplement: Supplementary Data [file supp_31_10_2780__index.html]

Improved Calibration of the Human Mitochondrial Clock Using Ancient Genomes — Improved Calibration of the Human Mitochondrial Clock Using Ancient Genomes — Supplementary Data 

# Improved Calibration of the Human Mitochondrial Clock Using Ancient Genomes

## Supplementary Data

files

**Files in this Data Supplement:**

- Supplementary Data - docx file
- Supplementary Data - doc file
- Supplementary Data - docx file
- Supplementary Data - xlsx file
- Supplementary Data - txt file
