# Supplementary material for: Improved Calibration of the Human Mitochondrial Clock Using Ancient Genomes
Source: Mol Biol Evol. 2014 Aug 5;31(10):2780–92. doi: 10.1093/molbev/msu222 (PMC4166928; doi:10.1093/molbev/msu222)
Supplement: Supplementary Data [file supp_msu222_Rieux_et_al_MBE-14-0276-Supplemental_materials.docx]

**Appendix S1. Library preparation and data processing**

Molecular processing

Approximately 200 ng of genomic DNA from each sample was sheared by sonication using a Bioruptor system (Diogenode) and used to construct a double-indexed Illumina sequencing library, with barcodes specific for each sample, as described in Kircher et al. (2012). Libraries were then pooled in equimolar ratio and mtDNA sequences were enriched via in-solution based hybridization-capture method (Maricic et al. 2010). The hybridization eluate was measured by qPCR and then amplified to produce a final concentration of 10 nmol. Samples were thus sequenced on a Solexa GAII lane using a paired end 75 cycles run, plus two 7nt indexes reads.

Data processing

In each Solexa GAII lane, 1% PhiX174 phage DNA was spiked in and used as a training set to estimate base quality scores with the IBIS base-caller. Reads with more than five bases having a PHRED scaled quality score below Q15 were discarded, as were reads having a single base quality in the index read (7nt) score below Q10. Reads with no mismatches to the expected double index sequences were assigned to each individual sample library and were then mapped to the revised mtDNA reference sequence (GenBank number: NC_012920) using MIA, an in-house assembler described in Briggs et al. (2009).

**Appendix S2. List and details on sequences used in this study**

A complete and detailed list of the samples/sequences used in that study can be found in the file S2.xls.

**Figure S2.** Geographical origin of all samples used in the study

**Appendix S3. Ancient samples considered in this study**

**Table S3.** Information on the ancient genomes used in this study

^*^ aAH: archaic ancient humans (Neanderthals) / aAMH: ancient anatomically modern humans

^**^ Used or not in the phylogenetic analyses

^***^ See skinner et al. (2005) for details on ESR dating of that sample

**Appendix S4. Details on internal node calibration points**

**Table S4.** Colonization/migration events considered for node calibration points. For the Americas and Postglacial expansion, each of the multiple haplogroups was independently constraint and the final date was estimated as the average of their coalescence times.


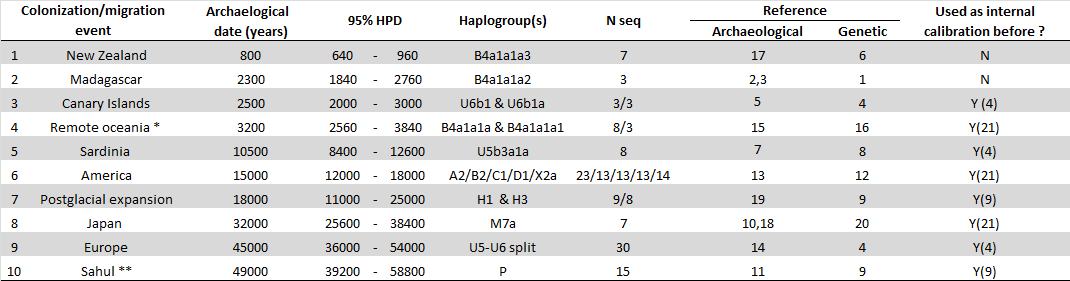


* Vanuatu, Tonga, Samoa, Cook Islands

** Australia + Papua New Guinea

References links (see page 14 of supplemental for full references):

1- Razafindrazaka et al. (2010), 2- Dewar et al. (2013), 3- Burney et al. (2004), 4- Pereira. (2010), 5- Navarro (1997), 6- Benton et al. (2012), 7- Malone (2003), 8- Calo et al. (2008), 9- Endicott & Ho (2008), 10- Takamiya (1996), 11- O'Connell & Allen (2004), 12- Fagundes et al. (2008), 13- Waters & Wier Stafford (2013), 14- Davies (2001), 15- Bedford et al. (2006), 16- Soares et al. (2011), 17- Kirch (2001), 18- Glover (1980), 19- Gamble et al. (2004), 20- Tanaka et al. (2004), 21- Henn et al. (2009)

**Appendix S5. Sequence composition**

**Table S5.** Base frequencies for contemporary (CH), ancient anatomically modern humans (aAMH) and ancient archaic humans (aAH) complete mtDNA sequences

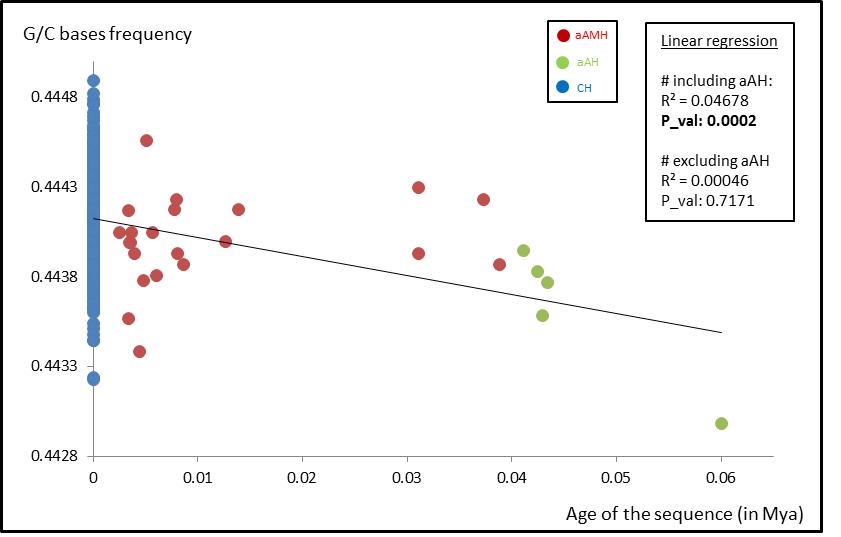


**Figure S5:** Temporal evolution of GC base frequency

**Appendix S6. Testing for damage patterns signal in ancient sequences**

**Table S6.** Expected vs. observed numbers of AT and GC singleton SNPs


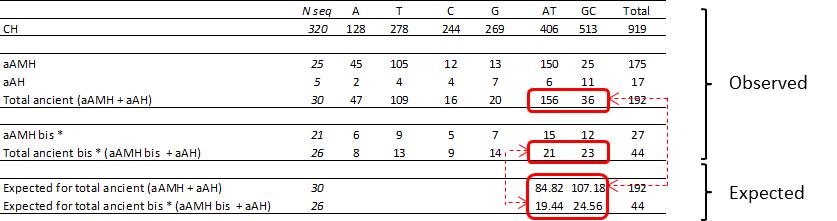


* Isolates ID: Ire8, Ajv52, Ajv70, Gok4. See Appendix S3 for details on those sequences.

1973 SNPs were called from the alignment including all the sequences but the chimpanzee one. Among the bi-allelic SNPs, we specifically investigated patterns of singleton SNPs to detect any signal of damage in ancient sequences.

When considering all the 30 ancient sequences, we observed 156 and 36 singleton SNPs at sites A/T and G/C respectively. We then calculated the number of such singletons SNPs that we would expect under the assumption of “no deamination” as follows:

N s-SNP-_AT_ expected = frequency of AT singleton in contemporary sequences x total number of observed singletons in ancient sequences

This translates in:

N s-SNP-_AT_ expected = (406/919)*192 = 84.82

And for GC singletons:

N s-SNP-_GC_ expected = (513/919)*192 = 107.18

The expected values for AT and GC singletons are significantly and smaller and higher respectively than the observed ones, thus suggesting deamination patterns in our ancient sequences pane. When excluding 4 ancient sequences (Ire8, Ajv52, Ajv70, Gok4), numbers of A/T singleton SNPS were not different from expectations computed assuming no deamination. On this basis, we excluded the four ancient Swedish sequences from all subsequent analyses.

**Appendix S7. Partitioning the mtDNA molecule**

**Table S7.** Optimal partitioning scheme and best-fit nucleotide substitution model for each partition of the mtDNA molecule as estimated with the software PartitionFinder. A) Best scheme partitioning obtained with PartitionFinder. Composition, number of sites and best model are given for each of the 4 partitions. B) Best model estimation for the whole mtDNA molecule.


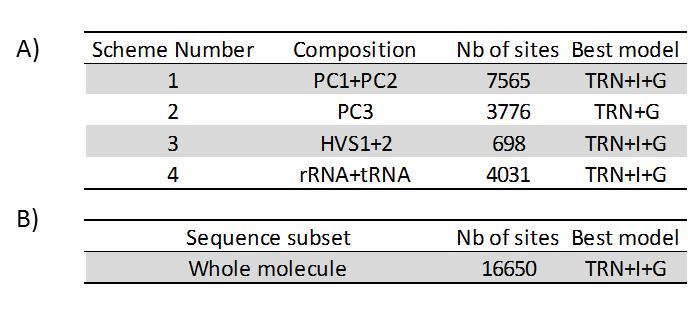


**Appendix S8. Date randomization analysis results**


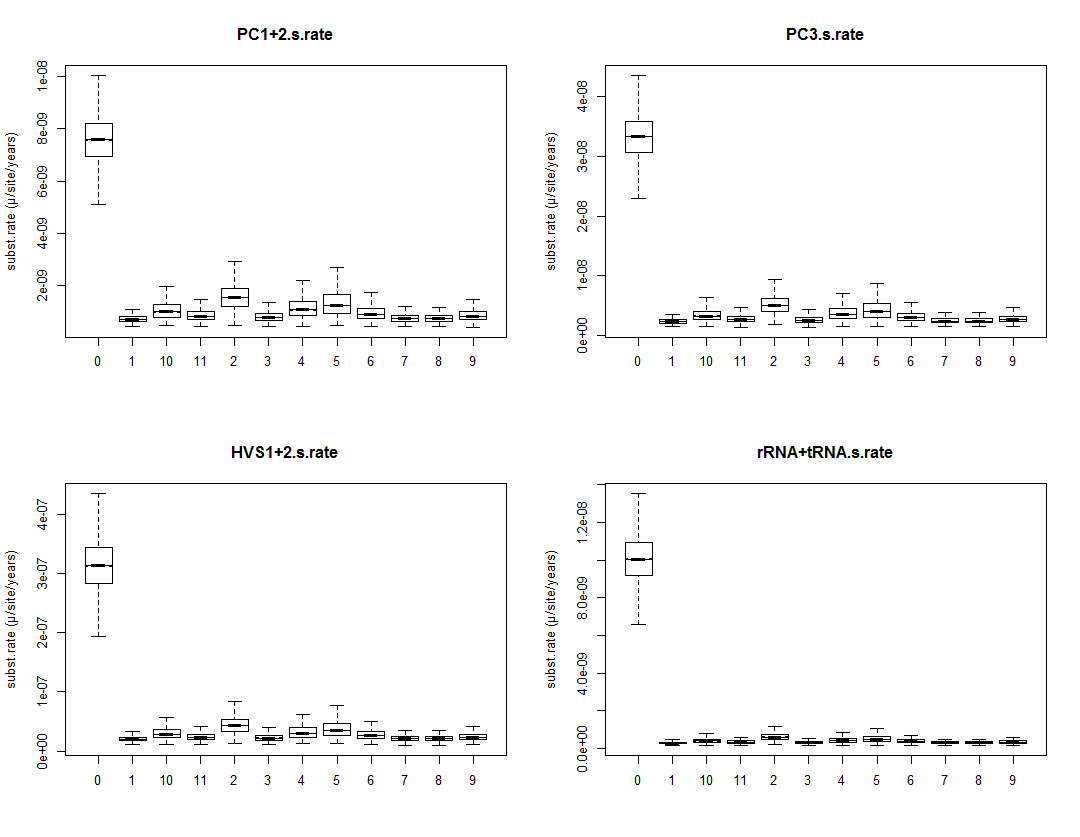


**Figure S8.** Substitution rates estimated on both the real and date-randomized data set considering different partition of the mtDNA molecule. 0 corresponds to the real dataset; 1-10 to the ten randomized datasets.

**Appendix S9. Complete tree based on 320 AMH, 21 aAMH, 5 aAH and one chimpanzee sequences**

The tree is too large to be shown here, it can be found (and displayed using any tree viewer) in nexus format in the file **S9.tree**.

**Appendix S10. Correlation between root-to-tip distances and age of the sequences**


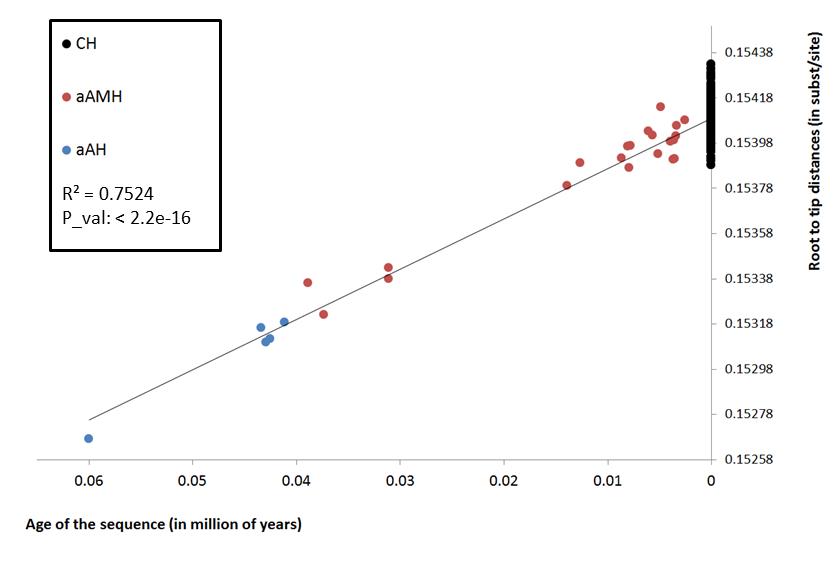


**Figure S10.** Relation between the age of the sequences and the root-to-tip distances extrapolated from the best tree (AppendixS9)

**Appendix S11. TMRCA of major haplogroups**

**Table S11**. Estimated age of the major haplogroups in our dataset assuming tips calibration based on all ancient samples

**Appendix S12. Rate and divergent time estimates obtained assuming different calibration scenarios**

**Table S12.** Bayesian estimates for divergence times, substitution rates and deviation to the strict clock obtained assuming various calibration scenario

**Figure S12.** Box plot (minimum, quartiles, median, and maximum) for Bayesian estimates of the whole mtDNA substitution rate (in µ/site/year) obtained under various calibration scenarios.

**Appendix S13. Estimation of whole mtDNA substitution rates performed using individual internal nodes and tips**

**Table S13**. Whole mtDNA substitution rate estimates performed using single tip and node calibrations.


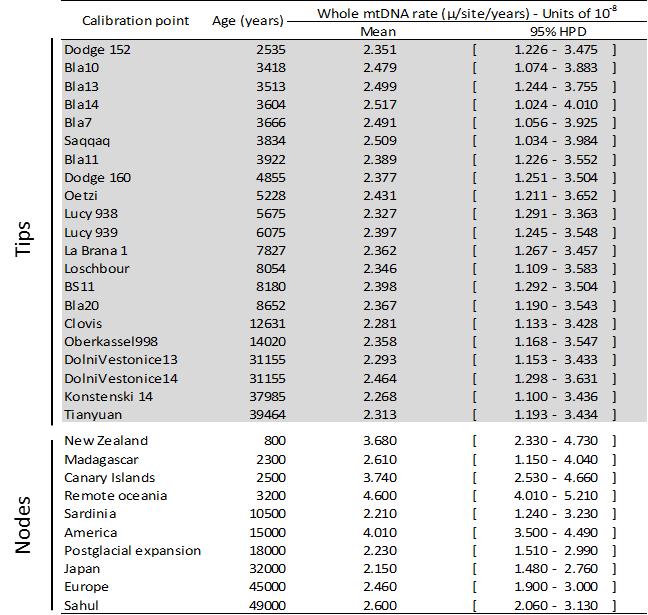

**Figure S13**. Relation between the estimated substitution rate at the whole molecule and the age of the calibration point (Panel A for tips based calibrations and Panel B for nodes based calibrations).

**Appendix S14. Comparison between the archaeological evidence and the estimated TMRCA of haplogroups for various colonization/migration events**

**Figure S14**. Comparison between the archaeological evidence (red shading, see Appendix S4 for the dates) and the estimated 95%HPD values of TMRCA for the various haplogroups involved in each of the ten colonization/migration events considered (see Table 2 for the values). For six out of ten of the events considered (Postglacial expansion, Sahul, Sardinia, Japan, Madagascar & Europe settlement), we observed 95% age HPD distributions of haplogroups overlapping with the archaeological dates. However, there was no overlap for the Canary Islands, Remote Oceania and New Zealand. In the case of the Americas, the 95% HPD distribution for two out of the five Native American haplogroups (B2 and X2a) overlapped with the archaeological record, but this was not the case for haplogroups A2, C1 & D1 as well as for the mean distribution calculated over the five haplogroups

**References**

1. Bedford, S., Spriggs, M., and Regenvanu, R. (2006). The Teouma Lapita site and the early human settlement of the Pacific Islands. Antiquity 80, 812-828.

2. Benton, M., Macartney-Coxson, D., Eccles, D., Griffiths, L., Chambers, G., and Lea, R. (2012). Complete Mitochondrial Genome Sequencing Reveals Novel Haplotypes in a Polynesian Population. Plos One 7.

3. Bollongino, R., Nehlich, O., Richards, M.P., Orschiedt, J., Thomas, M.G., Sell, C., Fajkosova, Z., Powell, A., and Burger, J. (2013). 2000 Years of Parallel Societies in Stone Age Central Europe. Science 342, 479-481.

4. Briggs, A.W., Good, J.M., Green, R.E., Krause, J., Maricic, T., Stenzel, U., Lalueza-Fox, C., Rudan, P., Brajkovic, D., Kucan, Z., et al. (2009). Targeted Retrieval and Analysis of Five Neandertal mtDNA Genomes. Science 325, 318-321.

5. Burney, D.A., Burney, L.P., Godfrey, L.R., Jungers, W.L., Goodman, S.M., Wright, H.T., and Jull, A.J.T. (2004). A chronology for late prehistoric Madagascar. Journal of Human Evolution 47, 25-63.

6. Calo, C., Melis, A., Vona, G., and Piras, I. (2008). Sardinian Population (Italy): a Genetic Review. International Journal of Modern Antropology 1, 1-121.

7. Cui, Y., Lindo, J., Hughes, C.E., Johnson, J.W., Hernandez, A.G., Kemp, B.M., Ma, J., Cunningham, R., Petzelt, B., Mitchell, J., et al. (2013). Ancient DNA Analysis of Mid-Holocene Individuals from the Northwest Coast of North America Reveals Different Evolutionary Paths for Mitogenomes. Plos One 8.

8. Davies, S.W.G. (2001). A very model of a modern human industry: new perspectives on the origins and spread of the Aurignacian in Europe Proceedings of the Prehistoric Society 67, 195-217.

9. Dewar, R.E., Radimilahy, C., Wright, H.T., Jacobs, Z., Kelly, G.O., and Berna, F. (2013). Stone tools and foraging in northern Madagascar challenge Holocene extinction models. Proceedings of the National Academy of Sciences of the United States of America 110, 12583-12588.

10. Endicott, P., and Ho, S.Y.W. (2008). A Bayesian evaluation of human mitochondrial substitution rates. American Journal of Human Genetics 82, 895-902.

11. Ermini, L., Olivieri, C., Rizzi, E., Corti, G., Bonnal, R., Soares, P., Luciani, S., Marota, I., De Bellis, G., Richards, M.B., et al. (2008). Complete Mitochondrial Genome Sequence of the Tyrolean Iceman. Current Biology 18, 1687-1693.

12. Fagundes, N.J.R., Kanitz, R., Eckert, R., Valls, A.C.S., Bogo, M.R., Salzano, F.M., Smith, D.G., Silva, W.A., Zago, M.A., Ribeiro-dos-Santos, A.K., et al. (2008). Mitochondrial population genomics supports a single pre-Clovis origin with a coastal route for the peopling of the Americas. American Journal of Human Genetics 82, 583-592.

13. Fu, Q., Meyer, M., Gao, X., Stenzel, U., Burbano, H.A., Kelso, J., and Paeaebo, S. (2013). DNA analysis of an early modern human from Tianyuan Cave, China. Proceedings of the National Academy of Sciences of the United States of America 110, 2223-2227.

14. Fu, Q., Mittnik, A., Johnson, P.L.F., Bos, K., Lari, M., Bollongino, R., Sun, C., Giemsch, L., Schmitz, R., Burger, J., et al. (2013). A Revised Timescale for Human Evolution Based on Ancient Mitochondrial Genomes. Current Biology 23, 553-559.

15. Gamble, C., Davies, W., Pettitt, P., and Richards, M. (2004). Climate change and evolving human diversity in Europe during the last glacial. Royal Society Philosophical Transactions Biological Sciences 359, 243-254.

16. Gilbert, M.T.P., Kivisild, T., Gronnow, B., Andersen, P.K., Metspalu, E., Reidla, M., Tamm, E., Axelsson, E., Gotherstrom, A., Campos, P.F., et al. (2008). Paleo-Eskimo mtDNA genome reveals matrilineal discontinuity in Greenland. Science 320, 1787-1789.

17. Glover, I.C. (1980). Agricultural origins in East Asia. In The Cambridge encyclopedia of archaeology.

18. Green, R.E., Malaspinas, A.-S., Krause, J., Briggs, A.W., Johnson, P.L.F., Uhler, C., Meyer, M., Good, J.M., Maricic, T., Stenzel, U., et al. (2008). A complete neandertal mitochondrial genome sequence determined by high-throughput Sequencing. Cell 134, 416-426.

19. Henn, B.M., Gignoux, C.R., Feldman, M.W., and Mountain, J.L. (2009). Characterizing the Time Dependency of Human Mitochondrial DNA Mutation Rate Estimates. Molecular Biology and Evolution 26, 217-230.

20. Kirch, P. (2001). On the Road of the Winds: An Archaeological History of the Pacific Islands Before European Contact.

21. Kircher, M., Sawyer, S., and Meyer, M. (2012). Double indexing overcomes inaccuracies in multiplex sequencing on the Illumina platform. Nucleic Acids Research 40.

22. Krause, J., Briggs, A.W., Kircher, M., Maricic, T., Zwyns, N., Derevianko, A., and Paeaebo, S. (2010). A Complete mtDNA Genome of an Early Modern Human from Kostenki, Russia. Current Biology 20, 231-236.

23. Malone, C. (2003). The Italian Neolithic: A synthesis of research. Journal of World Prehistory 17, 235-312.

24. Maricic, T., Whitten, M., and Paeaebo, S. (2010). Multiplexed DNA Sequence Capture of Mitochondrial Genomes Using PCR Products. Plos One 5.

25. Navarro, J. (1997). Pasado y presente de la arqueologı´a canaria. Espacio, Tiempo y Forma. Prehistoria y Arqueologı´a Canaria 10, 447– 478.

26. O'Connell, J.F., and Allen, J. (2004). Dating the colonization of Sahul (Pleistocene Australia-New Guinea): a review of recent research. Journal of Archaeological Science 31, 835-853.

27. Pereira, L., Silva, N.M., Franco-Duarte, R., Fernandes, V., Pereira, J.B., Costa, M.D., Martins, H., Soares, P., Behar, D.M., Richards, M.B., et al. (2010). Population expansion in the North African Late Pleistocene signalled by mitochondrial DNA haplogroup U6. Bmc Evolutionary Biology 10.

28. Rasmussen, M., Anzick, S.L., Waters, M.R., Skoglund, P., DeGiorgio, M., Stafford Jr, T.W., Rasmussen, S., Moltke, I., Albrechtsen, A., Doyle, S.M., et al. (2014). The genome of a Late Pleistocene human from a Clovis burial site in western Montana. Nature 506, 225-229.

29. Razafindrazaka, H., Ricaut, F.-X., Cox, M.P., Mormina, M., Dugoujon, J.-M., Randriamarolaza, L.P., Guitard, E., Tonasso, L., Ludes, B., and Crubezy, E. (2010). Complete mitochondrial DNA sequences provide new insights into the Polynesian motif and the peopling of Madagascar. European Journal of Human Genetics 18, 575-581.

30. Sanchez-Ouinto, F., Schroeder, H., Ramirez, O., Avila-Arcos, M.C., Pybus, M., Olalde, I., Velazquez, A.M.V., Prada Marcos, M.E., Vidal Encinas, J.M., Bertranpetit, J., et al. (2012). Genomic Affinities of Two 7,000-Year-Old Iberian Hunter-Gatherers. Current Biology 22, 1494-1499.

31. Skoglund, P., Malmstrom, H., Raghavan, M., Stora, J., Hall, P., Willerslev, E., Gilbert, M.T.P., Gotherstrom, A., and Jakobsson, M. (2012). Origins and Genetic Legacy of Neolithic Farmers and Hunter-Gatherers in Europe. Science 336, 466-469.

32. Soares, P., Rito, T., Trejaut, J., Mormina, M., Hill, C., Tinkler-Hundal, E., Braid, M., Clarke, D.J., Loo, J.-H., Thomson, N., et al. (2011). Ancient Voyaging and Polynesian Origins. American Journal of Human Genetics 88, 239-247.

33. Takamiya, H. (1996). Initial colonization, and subsistence adaptation processes in the late prehistory of the island of Okinawa. Bulletin of the Indo-Pacific Prehistory Association 15.

34. Tanaka, M., Cabrera, V.M., Gonzalez, A.M., Larruga, J.M., Takeyasu, T., Fuku, N., Guo, L.J., Hirose, R., Fujita, Y., Kurata, M., et al. (2004). Mitochondrial genome variation in Eastern Asia and the peopling of Japan. Genome Research 14, 1832-1850.

35. Waters, M., and Wier Stafford, T. (2013). The First Americans: A Review of the Evidence for the Late-Pleistocene Peopling of the Americas.
