# Supplementary material for: Improved Calibration of the Human Mitochondrial Clock Using Ancient Genomes
Source: Mol Biol Evol. 2014 Aug 5;31(10):2780–92. doi: 10.1093/molbev/msu222 (PMC4166928; doi:10.1093/molbev/msu222)
Supplement: Supplementary Data [file supp_msu222_Rieux_et_al_MBE-14-0276.docx]

Answer to editor and reviewers

**Editors’ comments**

Your manuscript has now been seen by two expert reviewers. Both feel (and as do I) that the manuscript is significantly improved in the present version and that it will be of broad interest to the readers of MBE. Both reviewers have a few additional minor comments, and I am returning the manuscript to you so that you can decide whether they are also useful to improve the manuscript. I do not feel that any of the comments are major or that further major revisions are required and I do not anticipate that the manuscript will go back to review. Hopefully this can move forward quickly at this point. I look forward to receiving your (minor) revised manuscript. Please also ensure that the sequences have been submitted to the public databases and accession numbers have been incorporated into the revised version of the text.

AR *et al.*: We are grateful to the reviewers and yourself for those final constructive and insightful comments. Our revised manuscript addresses the main remarks and accession numbers for sequences have now been incorporated. Please find a point-by-point replay to all comments.

**Reviewer 1 comments:**

This is an excellent manuscript and the authors have addressed the concerns raised in my previous review. The manuscript deals with an important topic and the authors have conducted a good range of analyses. I think that it will be a nice contribution to the literature. I have a number of minor comments, including a few suggestions for additional references.

Page 3 line 54. Replace “evolutionary story” with “evolutionary history”?

AR *et al.*: Done

Page 3 line 64. Replace “timetable” with either “timescale” or “timeframe”.

AR *et al.*: Done

Page 3 lines 68-69. An appropriate citation for this statement would be the recent review by Hipsley and Muller (2014).

AR *et al.*: Indeed, the message delivered by this review perfectly fits with our statement. We now cite it in the revised version of our manuscript.

Page 7 lines 173-174. In the plot of root-to-tip distances against sequence age, how were the root-to-tip distances estimated? Were these based on a phylogram estimated using a non-clock method?

AR *et al.*: Root-to-tip distances were computed using the “distRoot” function of the adephylo R package from a tree reconstructed assuming a non-constant rate of the molecular clock (obtained using BEAST but same results were reached using the maximum likelihood algorithm RaxML).

Page 10 lines 260-262. The authors might wish to note that a similar result was obtained by Millar et al. (2008), who were unable to distinguish between the pedigree-based estimate and tip-dated estimate of rates in penguins. However, both estimates were much higher than the ‘phylogenetic’ rate based on fossil calibrations. The authors are entirely free to ignore this suggestion, because the Millar study dealt with a completely different organism.

AR *et al.*: This reference has now been included.

Page 11 line 294. Replace “for a series reasons” with “for a series of reasons”.

AR *et al.*: Typo corrected.

Page 12 lines 302-303. The authors should point out that the date-randomisation test was only done for the full data set, and not the data sets that included each of the ancient DNA sequences in turn.

AR *et al.*: This has been included in the corresponding section of the methods (line 542).

Page 13 lines 336-351. The authors might wish to refer to the study by Crandall et al. (2012), who recognised the problem of calibrating using nodes rather than demographic events. They tied the geological event to the timing of population expansion, rather than to a genetic coalescence event.

AR *et al.*: We are now citing this interesting piece of work we were not previously aware of.

Page 13 lines 352-355. The authors might wish to cite a recent study by Duchene et al. (2014). This study not only confirmed a pattern of time-dependent rates in viruses, but also confirmed the combined impacts of purifying selection and saturation.

AR *et al.*: Again we are grateful to the reviewer for mentioning this interesting paper we now cite.

Page 14 lines 368-370. The superior fit of a relaxed clock can also be due to purifying selection rather than positive selection. The impact of purifying selection would vary among human lineages, depending on population size.

AR *et al.*: We agree and have now included the following sentence: “Alternatively, this variation in evolutionary rates could stem from purifying selection on mtDNA being more effective in some human populations than others, due to differences is population size and past demography.” (lines 372-374)

Page 15 lines 393-401. Here the authors might wish to note the extremely close similarity between their estimate and that of Endicott et al. (2010), who dated the human-Neanderthal split at 407kya (315-506 kya). Endicott and colleagues calibrated their estimate using the human-chimpanzee split, but attempted to minimise the impact of purifying selection by using only third codon sites of the mitochondrial genome.

AR *et al.*: We now mention this close similarity in rate estimates.

**Reviewer 2 comments**

This is a vastly improved version and the authors have taken the time to carefully consider and implement the suggestions of the reviewers. I have only a handful of comments that can easily be dealt with.

1) The final sentence of the abstract "As such, estimates of mtDNA substitution rates based on dated tips should be considered as more reliable" is awkward and does not read well. I would consider eliminating it all together since the previous sentence sums up the paper nicely.

AR *et al.*: Rather than deleting it completely, we merged the two last sequences as following: “Our results demonstrate that, for the same dataset, estimates based on individual dated tips are far more consistent with each other than those based on nodes and should thus be considered as more reliable.”

2) I would eliminate the "for instance" on line 53.

AR *et al.*: Done

3) On line 113 I would avoid starting the sentence with a number.

AR *et al.*: Modified, it now reads: “A total of 1973 SNPs were….”

4) On line 117, it would be worth saying where the four sequences were from both in terms of geography and the paper they were published.

AR *et al.*: We now explicitly mention that this information is reported in Table S3.

5) On line 281, the tense in this sentence is awkard: "Altogether, these results point to tip calibration providing far more consistent estimates than internal node calibrations." How about 'these results strongly suggest that tip calibrations are far more consistent..." The following sentence could also be cleaned up.

AR *et al.*: Modified.

This is an excellent paper and I'm looking forward to seeing it in press.

AR *et al.*: Thanks you for this nice comment. We are also looking forward to seeing the paper in print.
