# Supplementary material for: Improved Calibration of the Human Mitochondrial Clock Using Ancient Genomes
Source: Mol Biol Evol. 2014 Aug 5;31(10):2780–92. doi: 10.1093/molbev/msu222 (PMC4166928; doi:10.1093/molbev/msu222)
Supplement: Supplementary Data [file supp_msu222_Rieux_et_al_MBE-14-0276.doc]

Research Department of Genetics, Evolution and Environment

July 2014, the 14^th^

Dear Editor,

Please find enclosed the final revision of our manuscript (MBE-14-0276-R1) entitled “Improved calibration of the human mitochondrial clock using ancient genomes”.

We followed all the reviewers’ recommendations and suggestions and also included the accession numbers of the new complete mitochondrial sequences.

We are looking forward to seeing our paper in print in Molecular Biology and Evolution and wish to thank you and the reviewers for the time they spent into improving our article.

Yours sincerely,

Adrien Rieux (on behalf of all co-authors)
